# ﻿A new digamasellid mite of the subgenus Longoseiulus Lindquist (Acari, Mesostigmata) from Slovakia

**DOI:** 10.3897/zookeys.1131.95246

**Published:** 2022-11-22

**Authors:** Peter Mašán

**Affiliations:** 1 Institute of Zoology, Slovak Academy of Sciences, Dúbravská cesta 9, 845-06 Bratislava, Slovakia Institute of Zoology, Slovak Academy of Sciences Bratislava Slovakia

**Keywords:** Description, Digamasellidae, *Fraxinus*, morphology, saproxylic habitat, systematics

## Abstract

A new digamasellid mite, Longoseius (Longoseiulus) disparisetus**sp. nov.**, was described from females found in the wood detritus of tree cavity of freshly felled elm (*Fraxinus* sp.) in a park in southwestern Slovakia. The new species differs from known congeners by the number of setae on some leg segments (genu II with eight setae, tibiae II and III with seven and six setae, respectively) and by the unusual presence of three pairs of conspicuously shortened setae (*J3*, *J4*, and *Z3*) on the posterior dorsal shield. In other known *Longoseiulus* species, the genu II has 11 setae, the tibiae II and III have 10 and seven setae, respectively, and almost all dorsal setae are of similar length (except for the elongated *Z4* and *S4*), none of which is formed as a microseta. A dichotomous key for females is provided to identify species classified worldwide in *Longoseiulus*.

## ﻿Introduction

*Longoseiulus* was originally described by Lindquist (1975) as a subgenus of Longoseius Chant, 1961 and later treated at the subgenus level in Longoseius by Hirschmann and Wiśniewski (1982) and Castilho et al. (2012), or *Dendrolaelaps* Halbert, 1915 by Shcherbak (1980) and Barilo (1989). Karg (1993) considered *Longoseiulus* to be a synonym of the valid genus *Longoseius*.

The concept of *Longoseiulus* adopted here is largely based on the diagnosis of Lindquist (1975) and mainly on the following characters: (1) leg III with four setae on trochanter instead of normally five setae, and with seven or fewer setae on genu and tibia instead of eight or nine setae; (2) basitarsi II and III each with two or three setae instead of normally four setae, basitarsus IV with one to three setae instead of normally three or four setae; (3) hypostomal furrow of gnathosoma with most proximal fifth row of denticles not distinctly wider than preceding rows (in other genera this fifth row of denticles is conspicuously wider than preceding rows, or six rows of denticles of similar width are rarely present); (4) four-toothed movable digit of female chelicerae; (5) setae *j2* transversely aligned with *j1* and *z1* on podonotal shield; (6) sclerotized anterior margin of opisthonotal shield with a deep double incision in the middle; (7) peritreme of adults and deutonymphs similar in length, shortened and extending at most only slightly beyond posterior margin of coxa II.

The subgenus Longoseiulus Lindquist, 1975 is a small group of digamasellid mites and currently includes only seven known species from Europe (*aberrans*, *longuloides*, *longulus*, *ornatus*), Asia (*nobilis*, *ornatosimilis*), and North America (*brachypoda*), which are almost always found in saproxylic habitats, especially in decomposing wood of various coniferous and broad-leaved deciduous trees, and in subcortical spaces associated with galleries of bark- and wood-boring beetles. Phoretic activity of deutonymphs is common in many xylophagous beetles such as Cerambycidae, Cleridae, Elateridae, Scolytinae, and Pyrochroidae (Hirschmann and Wiśniewski 1982).

The aim of this study is to describe a new species of the subgenus Longoseius (Longoseiulus) from Slovakia and thus to contribute to the knowledge of the fauna of Digamasellidae in Europe. This work is part of a project aimed at increasing our collective knowledge of the mite fauna of Slovakia. In this sense, the finding of the new species also represents a first record of the genus *Longoseius* for Slovakia.

## ﻿Materials and methods

**Figures 1, 2.**
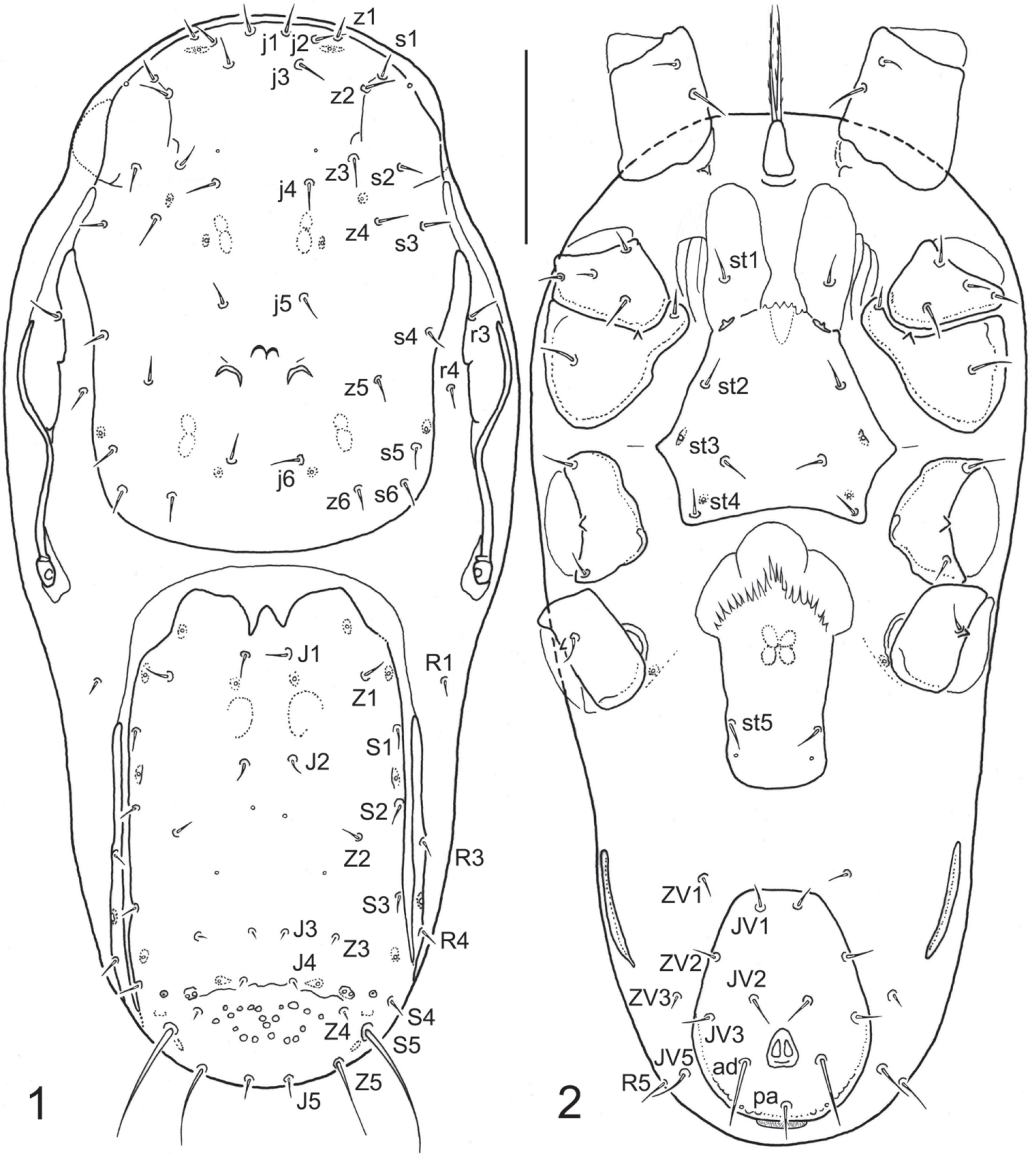
Longoseius (Longoseiulus) disparisetus sp. nov., female, with symbols for chaetotactic notation of idiosomal setae **1** dorsal idiosoma **2** ventral idiosoma. Scale bar: 50 µm.

Mites were extracted from decomposing wood detritus using a modified Berlese-Tullgren funnel equipped with a 40-W lamp and preserved in ethyl alcohol. For identification, the mites were mounted on slides with Swan’s medium (gum arabic/chloral hydrate). A Leica DM 1000 light microscope with a Leica EC3 digital camera was used for measurements and micrographs. The photomicrographs were processed using Adobe Photoshop Elements 8 software. Measurements were made on specimens mounted on a microscope slide. Idiosoma and shield lengths were measured along their midlines, and widths were measured at their widest point (unless otherwise noted in the description). The lengths of the ventral idiosomal shields are midline, from the anterior to the posterior margin of each structure, including the hyaline anterior extension of the epigynal shield and excluding the posterior cribrum of the anal shield. Legs were measured excluding the ambulacral apparatus. Setae were measured from the bases of their attachments to their tips. The dimensions of the structures are given as ranges (minimum to maximum). The number of teeth on the cheliceral digits does not include the apical hook. Setal notation symbols for the idiosoma follow Lindquist and Evans (1965), slightly modified by Lindquist (1994), and notation symbols for leg setae follow Evans (1963). Terminology for the other anatomical structures follows Evans and Till (1979). The chaetotaxy symbols used here are shown in Figs 1, 2.

## ﻿Results

### ﻿ Longoseius (Longoseiulus) disparisetus sp. nov.

Taxon classificationAnimaliaMesostigmataDigamasellidae

544FAE8D-656A-5D88-BEC8-56F13FB84CF3

https://zoobank.org/B759A60A-49EF-400D-8823-33B336AA41EF

Figs 1–2 , 3–7 , 8–10 , 11

#### Type material examined.

***Holotype* female**: SW Slovakia, Podunajská Rovina Flatland, Bratislava Capital, Petržalka Settlement, Sad Janka Kráľa Park (48°08'N, 17°06'E), elev. 135 m, 25 October 2020, wood detritus from a cavity in the trunk of an old and freshly felled elm (*Fraxinus* sp.), colonised by an unidentified ant species (Hymenoptera: Formicidae). ***Paratypes***: five females, with the same data as the holotype. The type material is deposited in the Institute of Zoology of the Slovak Academy of Sciences, Bratislava, Slovakia.

#### Diagnosis

**(female).** The presence of three pairs of microsetae (*J3*, *J4*, and *Z3*) on the posterior dorsal shield of the new species is unique and distinctly different from all other known species of the subgenus Longoseiulus. Some displacement of *J3* toward the bases of *J4*, making the bases of *J3* and *Z3* almost transversely aligned, also makes the idiosomal chaetotaxy of Longoseius (Longoseiulus) disparisetus sp. nov. peculiar. Most of the dorsal setae are of approximately equal length in all other congeneric species, with the exception of *Z5* and *S5*, which are conspicuously long in most representatives of the family and are located at the posterior margin of the opisthonotum.

There are other important diagnostic characters for this new species: (1) the absence of dorsal setae *r5* (these setae are present on the soft cuticle in females of the related species whose setae *Z3* are prominent and moderately elongate), (2) the absence of many leg setae that Lindquist (1975) indicated in his original definition as being present in *Longoseiulus* species, possibly based on the chaetotaxy of the type species Longoseius (Longoseiulus) longulus. There is one other species of *Longoseiulus* for which Hurlbutt (1967) originally reported the chaetotaxy of the legs, namely L. (L.) brachypoda. In comparison with the above species, the new species was found to have setal deficiencies in the following leg segments: genu II with eight instead of 11 setae, tibia II with seven instead of 10 setae, tibia III with six instead of seven setae, and telotarsi II and III with 11 instead of 12 setae. For a further comparison of the chaetotaxy of the legs of the new species with those of the subgenus Longoseius, see Table 1.

**Table 1.** Number of setae on selected leg segments of *Longoseius* [based on Hurlbutt (1967), Lindquist (1975) and own data]. Explanations: * – new information for a diagnosis of the subgenus Longoseiulus, ** – new information for a diagnosis of the genus *Longoseius*.

Leg segment	Taxon	Number of setae			
		Leg I	Leg II	Leg III	Leg IV
**Femur**	subgen. Longoseius	10	10	5	6
	*disparisetus* sp. nov.	12	10	6	6
	subgen. Longoseiulus	12–13	10	6	6
**Genu**	subgen. Longoseius	8	5	4	3
	*disparisetus* sp. nov.	11	8*	7	7
	subgen. Longoseiulus	11–12	11	7	7–8
**Tibia**	subgen. Longoseius	9	7	7	7
	*disparisetus* sp. nov.	12	7*	6**	7
	subgen. Longoseiulus	12	10	7	7
**Telotarsus**	subgen. Longoseius	–	11	10	10
	*disparisetus* sp. nov.	–	11*	11*	12
	subgen. Longoseiulus	–	12	12	12

#### Description

**(female). *Dorsal idiosoma*** (Figs 1, 8). Idiosoma 310–335 μm long and 140–155 μm wide (six measured specimens), narrowly oval, only moderately elongate, rounded anteriorly and posteriorly, suboval, widest in anterior part, at level of anterior ends of peritremes. Dorsal shield completely divided into podonotal and opisthonotal parts, not completely covering the dorsal surface, exposing narrow strips of lateral soft cuticle. Podonotal shield 153–167 μm long and 106–121 μm wide, anteriorly and posteriorly broadly rounded, with smooth and unornamented surface (not considering sigillae, sclerotic nodules, and some fine and very short lines on anterolateral areas), 18 pairs of setae (*j1*–*j6*, *z1*–*z6*, *s1*–*s6*) and two pairs of usually crescent-shaped subsurface sclerotic nodules between setae *z5* (the outer pair with larger and more conspicuous nodules than the inner pair of contiguous nodules arranged anteriorly). Two pairs of anterior marginal setae present, namely *r3* on peritrematal shields and *r4* on soft cuticle between podonotum and peritrematal shields. Opisthonotal shield 147–166 μm long and 76–93 μm wide (excluding lateral strips of scutal elements), anteriorly and posteriorly broadly rounded, laterally straight and nearly parallel, largely smooth except for a small foveolate area between setae *Z4*, with 15 pairs of setae (*J1*–*J5*, *Z1*–*Z5*, *S1*–*S5*); anterior margin of well-sclerotized part of shield with two deep medial incisions, flanked by narrow band with nearly desclerotized margin. Four pairs of posterior marginal setae present: *R1* on soft cuticle adjacent to anterolateral margins of opisthonotum; *R3* and *R4* on narrow longitudinal bands of scutal elements parallel to lateral margins of opisthonotal shield and narrowly fused to posterolateral margins of shield; *R5* usually on soft integument on ventral side near setae *JV5* or rarely on margin of opisthonotal shield. All dorsal setae smooth and needle-like, usually similar in length; three pairs of setae (*J3*, *J4*, and *Z3*) conspicuously reduced in length and each formed as a microseta (2–4 μm long); *S5* longest (37–48 μm); lengths of other dorsal setae as follows: *j1*–*j6*, *z1*–*z6*, *s1*–*s6*, *r4*, *J1*, *J2*, *Z1*, *Z2*, and *S1*–*S4* = 7–11 μm; *J5*, *Z4*, *R1*, *R3*, and *R4* = 5–7 μm; *Z5* = 22–30 μm; *r3* and *R5* = 10–14 μm.

**Figures 3–7.**
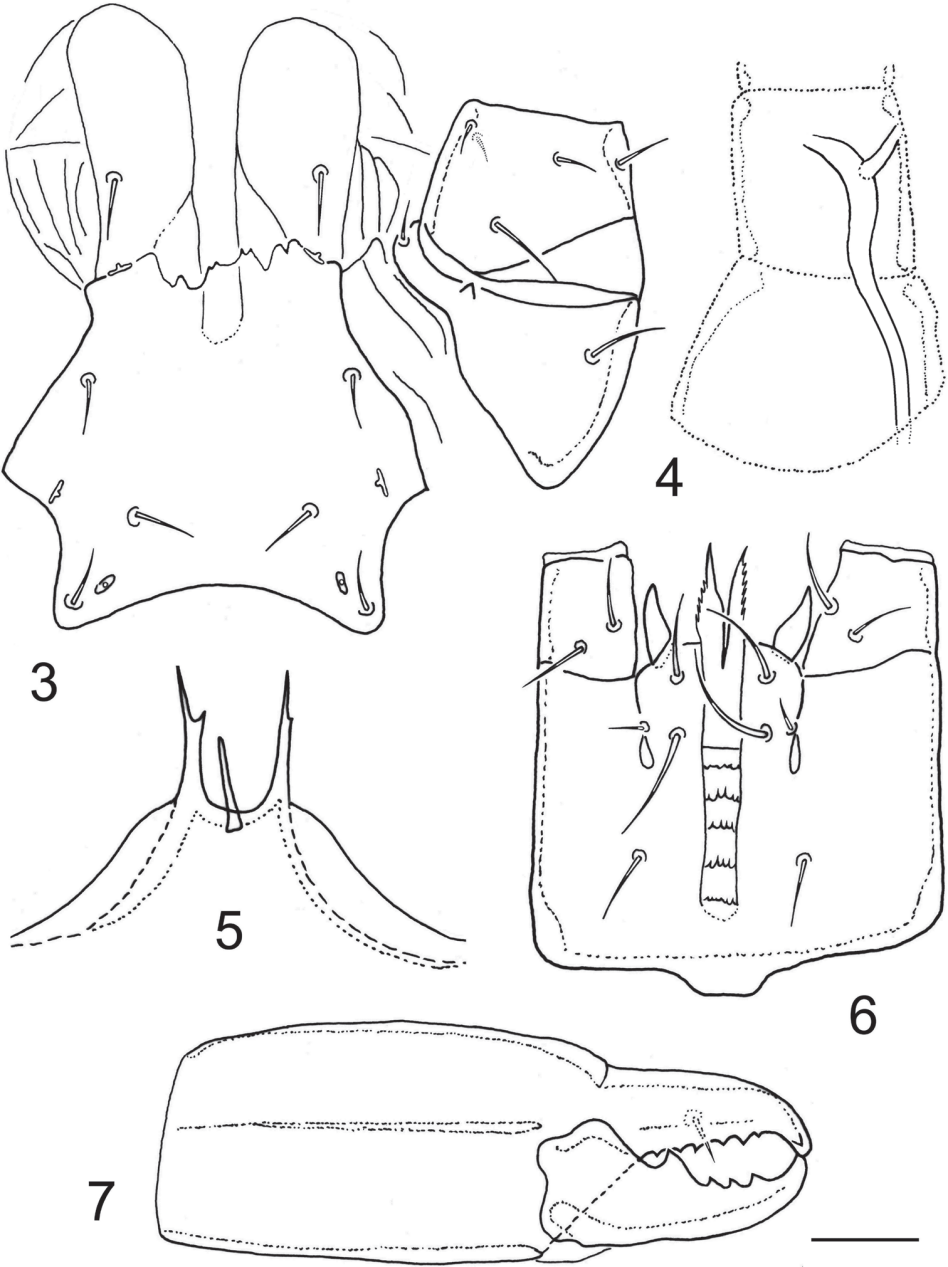
Longoseius (Longoseiulus) disparisetus sp. nov., female **3** sternal shield and adjacent coxa with trochanter of leg II **4** tubular part of sperm access system in proximal segments of leg III **5** epistome **6** gnathosoma, ventral view **7** chelicera, lateral view. Scale bar: 20 µm.

***Ventral idiosoma*** (Figs 2, 3, 9, 10). Tritosternum with short columnar base and two laciniae; laciniae divided to base, each sparsely, finely and shortly pilose. Sternal shield weakly sclerotized and defined (compared to epigynal and ventrianal shields), longer than wide, with two lobe-shaped anterior extensions each bearing a seta (*st1*), four pairs of sternal setae (*st1*–*st4*), and three pairs of poroidal structures; *st3* more closely spaced than the other pairs of sternal setae; posterior margin moderately concave and shaped into posterolateral angles, each bearing a metasternal seta (*st4*); shield smooth over entire surface, except for small desclerotized areas lateral to *st1*, each with three to five short lines (Figs 3, 9). Epigynal shield elongate, 81–93 μm long, widest at anterior hyaline part (48–55 μm), formed as convex and moderately trilobate marginal structure, narrowest at level of *st5* (27–33 μm), slightly rounded posteriorly, with a pair of genital setae (*st5*) near posterolateral margins and a pair of genital poroids posterior to *st5*. Exopodal and endopodal plates or platelets not developed, absent. Peritremes usually with dorsolateral to lateral position on idiosoma (Fig. 1), shortened, 67–84 μm long, each with anterior end extending slightly beyond posterior margin of coxa II. Peritrematal shield developed only along anterior part of peritreme (*r3* captured by shield), narrowly connected to podonotal shield at level of *s3*, completely reduced along posterior part of peritreme and weakly developed near stigma, with very short poststigmatic part. A pair of strongly elongated and longitudinally oriented metapodal platelets present; platelets narrow, 34–42 μm long and slightly curved. Ventrianal shield expanded posteriorly, vase-shaped, distinctly longer than wide (69–80 μm long and 49–58 μm wide), with nearly straight anterior margin, broadly rounded posterior margin, smooth surface, four pairs of preanal setae (*JV1*–*JV3*, *ZV2*) in addition to three circum-anal setae and one pair of gland pores located near posterior margin at level of postanal seta (*pa*); adanal setae (*ad*) at least twice as long as postanal seta (*ad* 18–25 μm, *pa* 8–12 μm); anus and cribrum relatively small. Soft opisthogastric cuticle with three pairs of preanal setae (*ZV1*, *ZV3*, and *JV5*). Ventral setae similar in form to those on dorsal side of idiosoma, with the following lengths: *st1*–*st5*, *JV1*–*JV3*, *ZV1* and *ZV2* = 8–11 μm, *ZV3* = 5–7 μm, *JV5* = 12–16 μm.

***Sperm induction system*** (Fig. 4). Sperm duct relatively well sclerotized, long and wide, located within the coxa, trochanter, and femur of legs III, apparently bifurcate near its terminal part, and opened at level of distal part of femur III.

***Gnathosomal structures*** (Figs 5–7). Epistome triramous; median process short, thin, straight, usually with obtuse tip; lateral rami conspicuously longer and thicker than median process, each sharply pointed and usually with a small subapical denticle or tine (Fig. 5). Hypostomal furrow relatively narrow, with five transverse rows of denticles connected laterally by a line; all transverse rows of denticles uniformly narrow and fifth (most proximal) row not noticeably wider than preceding rows, each row with few (3–7) sparsely to regularly spaced denticles; corniculi horn-like and divergent; internal malae extending beyond corniculi and formed as pointed projections with serrated outer margins (Fig. 6). Subcapitular setae smooth and needle-like, *h2* shortest and *h3* longest. Palptrochanter with five setae, palptarsal apotele two-tined. Chelicerae relatively large (compared to size of gnathosoma or idiosoma), with middle article 68–78 μm long; cheliceral digits dentate, similar in size; movable digit quadridentate, with most proximal tooth largest; fixed digit with 5–8 teeth in addition to terminal hook bearing small subapical tooth, and with minute setiform *pilus dentilis* (Fig. 7); a coronet-like fringe, dorsal cheliceral seta, and antiaxial lyrifissure not discernible.

**Figures 8–10.**
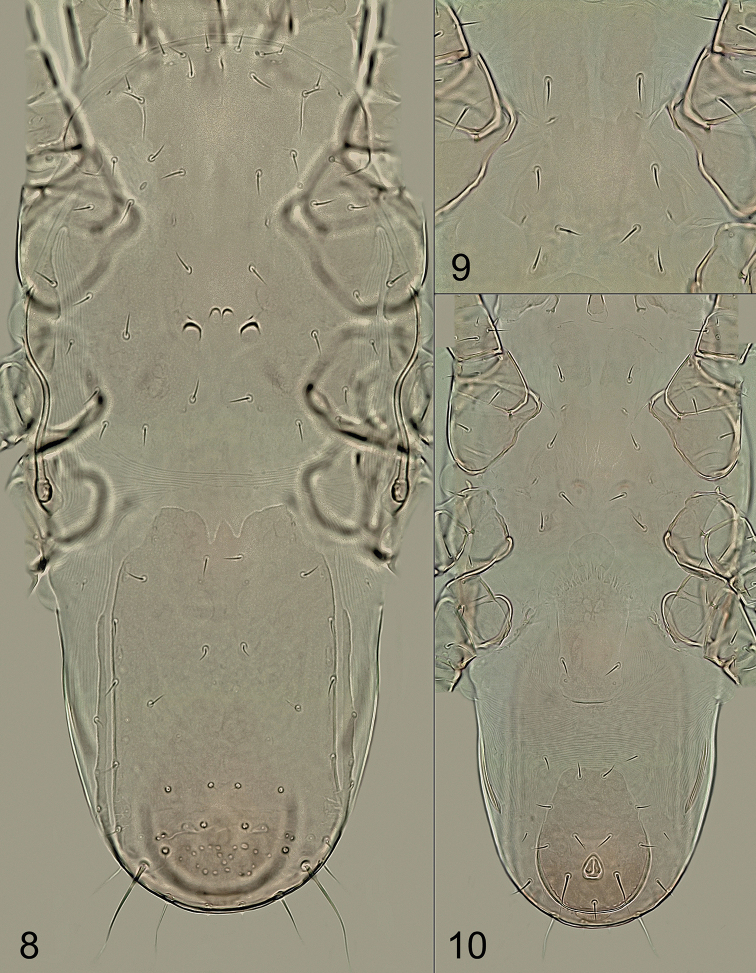
Longoseius (Longoseiulus) disparisetus sp. nov., photomicrographs of female **8** dorsal idiosoma **9** sternal region **10** ventral idiosoma. Not to scale.

**Figure 11.**
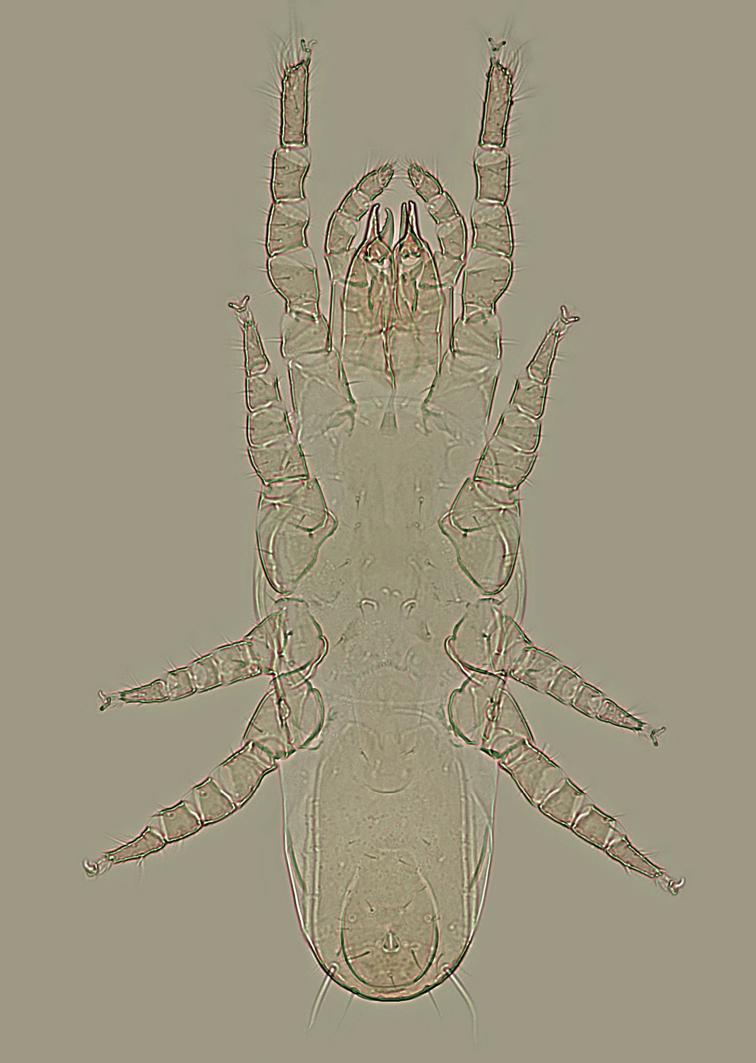
Longoseius (Longoseiulus) disparisetus sp. nov., dorso-ventral habitus of female (photomicrograph). Not to scale.

***Legs*** (Fig. 11). All legs with well-developed pretarsus and ambulacral apparatus (including pulvillus and two claws), distinctly shorter than idiosoma: legs I 180–205 μm, legs II 135–155 μm, legs III 105–120 μm, and legs IV 135–160 μm long. Leg segments not spurred ventrally, with smooth and needle-like setae, except telotarsi II–IV with setae *ad1*, *pd1*, *av1*, *pv1*, and *md* (when present) shortened and thickened, spine-like, and *pd2* thickened. Chaetotactic formulae for each leg segment as follows: leg I – coxa (2), trochanter (6), femur 2-3/1, 2/2-2 (12), genu 2-2/1, 2/2-2 (11), tibia 2-2/1, 3/2-2 (12); leg II – coxa (2), trochanter (5), femur 2-2/1, 1/2-2 (10), genu 1-2/1, 2/1-1 (8), tibia 1-1/1, 2/1-1 (7); leg III – coxa (2), trochanter (4), femur 1-2/1, 1/0-1 (6), genu 1-2/1, 2/0-1 (7), tibia 1-1/1, 2/1-0 (6); leg IV – coxa (1), trochanter (5), femur 1-2/0, 1/1-1 (6), genu 1-2/1, 2/0-1 (7), tibia 1-1/1, 2/1-1 (7). Tibia I without one anterodorsal seta (*ad3*), tibia III without both posterolateral setae (*pl1*, *pl2*), and genua III and IV each with only one ventral seta (*pv* absent). Basitarsi of legs II and III each with two dorsal setae (*al3* and *pl3* absent), basitarsus IV with only one dorsal seta (*al3*, *pd3*, and *pl3* absent). Setation of telotarsi II-III-IV, respectively, 11-11-12 (excluding the pair of dorsodistal seta-like processes); telotarsi II and III each lacking a mediodorsal seta (*md*), but telotarsus IV with seta *md* present.

#### Taxonomic note.

Among the closest relatives with known chaetotaxy of the legs, the new species is easily recognised by the specific number of setae on several leg segments (see Table 1 and the above diagnosis). It lacks many leg setae that Lindquist (1975) indicated in his original definition as being present in *Longoseiulus* species, namely *al*, *ad3*, *pl* on the genu II; *al*, *ad2*, *pl* on the tibia II; *pl* on the tibia III; and *md* on the telotarsi II and III. This requires some amendments to the diagnosis introduced by Lindquist (1975) for *Longoseiulus* and partially for the genus *Longoseius* (a pl seta found on the tibia III in *Longoseiuscuniculus* Chant, 1961 is absent in the new species).

#### Etymology.

The specific name is derived from the Latin words *dispār* (unequal or dissimilar) and *sēta* (bristle or hair) and refers to the striking differences in length between the setae on the opisthonotal shield of the female of this new mite (three pairs of setae are greatly reduced and formed as microsetae).

##### ﻿Key to the worldwide species of the subgenus Longoseiulus (females)

*Longoseiulus* includes seven described species, all known from the Holarctic. Only Longoseius (Longoseiulus) aberrans Hirschmann, 1960 is not included in the following key because its description is based solely on the male stage (it is one of the species with 21 pairs of setae on the anterior dorsal surface, including *r5*). It is not possible to reliably subdivide the individual species of *Longoseiulus* known to date based on literature data alone, without examining the type specimens. They should be thoroughly revised, redescribed, and compared in future studies to obtain a more accurate and reliable identification key than the one presented in this study. The original descriptions of most *Longoseiulus* species were not elaborated with the necessary precision (for example, they lack information on the chaetotaxy of the legs or on the measurement of important setal or scutal structures). Therefore, it is not currently possible to define and delimit some species morphologically on the basis of reliable characters. It is likely that a future revision will reveal the conspecificity of some species now placed in this subgenus.

**Table:** 

1	Anterior dorsum with 20 pairs of setae, including two pairs of marginal setae on peritrematal shields (*r3*) or soft cuticle (*r4*); setae *Z3* short (*Z3*≤*Z2*, *Z3*≤*j5*), never reaching beyond posterolateral margins of opisthonotal shield; idiosoma relatively small, 290–335 µm long	**2**
–	Anterior dorsum with 21 pairs of setae, including three pairs of marginal setae on peritrematal shields (*r3*) or soft cuticle (*r4*, *r5*); setae Z3 long (*Z3*≥2×*Z2*, *Z3*≥2×*j5*), reaching beyond posterolateral margins of opisthonotal shield; idiosoma relatively large, 330–390 µm long	**3**
2.	Three pairs of opisthonotal setae (*J3*, *J4*, and *Z3*) conspicuously shortened, formed as microsetae (2–4 µm long), at least twice shorter than other dorsal setae; inner pair of sclerotic nodules on hexagonal dorsal area with anterior position as outer pair; tibia III with 6 setae (without posterolateral setae)	**Longoseius (Longoseiulus) disparisetus sp. nov.** [Slovakia]
–	Three pairs of opisthonotal setae (*J3*, *J4*, and *Z3*) never shortened and almost as long as other dorsal setae (except *Z5* and *S5*); two pairs of sclerotic nodules on hexagonal dorsal area in a transverse row; tibia III with 7 setae (with one posterolateral seta)	**L. (L.) longulus (Hirschmann, 1960)** [Germany]. **L. (L.) longuloides Hirschmann & Wiśniewski, 1982** [Ukraine]
3	Ventrianal shield with three pairs of preanal setae (*JV1*, *JV2*, and *ZV2*)	**L. (L.) ornatus (Hirschmann, 1960)** [Germany]
–	Ventrianal shield with four pairs of preanal setae (*JV1*–*JV3* and *ZV2*)	**L. (L.) brachypoda (Hurlbutt, 1967)** [U.S.A., Louisiana]. **L. (L.) ornatosimilis (Shcherbak, 1980)** [Russia, Buryat]. **L. (L.) nobilis (Barilo, 1989)** [Uzbekistan]

## Supplementary Material

XML Treatment for Longoseius (Longoseiulus) disparisetus
